# Impact of Virtual Reality on Pharmacology Education: A Pilot Study

**DOI:** 10.7759/cureus.43411

**Published:** 2023-08-13

**Authors:** Kevin Kim, Nicholas Xie, Leslie Hammersmith, Yerko Berrocal, Monzurul A Roni

**Affiliations:** 1 Health Sciences Education and Pathology, University of Illinois Chicago, Peoria, USA; 2 Foundational Sciences, Alice L. Walton School of Medicine, Bentonville, USA

**Keywords:** interactive, three-dimensional, medical education, pharmacology, virtual reality

## Abstract

Introduction

Virtual reality (VR) is a powerful tool in health professional education. It has been successfully implemented in various domains of education with positive learning outcomes. The three-dimensional (3D) visualization offered by VR can potentially be applied to learn complex pharmacology topics. This study aims to investigate whether VR technology can improve the learning of complex pharmacological concepts.

Methods

A VR learning module on cardiovascular drugs was developed using Kern’s six-step framework. 32 medical students participated in the pilot study. Their pharmacology knowledge was assessed using pre- and post-intervention tests. Additionally, feedback from the participants were collected through a post-intervention survey that assessed learner satisfaction, ease of use, perceived usefulness, quality of visual elements, intention to use, and comfort level during the VR experience.

Results

Participants scored significantly higher in the post-intervention test than in the pre-intervention test (p <0.05). A majority of the participants (90%) were satisfied with the VR module, finding it easy to use, and time efficient. A minority of participants (15%) preferred a traditional learning format while some participants (20%) experienced discomfort in VR.

Conclusion

Our findings suggest that VR enhances pharmacology knowledge in medical students and is well-received as an innovative educational tool. By providing immersive 3D visualization of complex drug actions, VR has the potential to transform pharmacology education into an engaging and effective learning experience.

## Introduction

Pharmacology is one of the core disciplines of health professional education. Traditionally, it has been taught by dedicated lectures or case-based sessions [1]. However, the conventional pedagogical approaches employed in pharmacology education are considered among the least effective for a discipline that involves learning an ever-expanding field of pharmacological agents [2]. New strategies, such as interactive learning in a three-dimensional (3D) virtual environment, appear to be more effective, particularly in teaching complex pharmacological concepts like drug and protein interactions [3].

In recent years, virtual reality (VR) technology has proven successful in various areas of health professional education, including anatomy and clinical skills. For example, VR is often used in the creation of intricate 3D models depicting anatomical structures. VR facilitates the virtual manipulation of anatomical components, offering a heightened degree of visualization. Furthermore, the successful utilization of VR extends to the realm of simulating clinical procedures such as training of laparoscopic colorectal surgery [4-7]. VR is a powerful tool that allows participants to learn by immersing in and manipulating a 3D environment facilitated by a head-mounted display (HMD) or VR headset and handheld controllers. The high-fidelity graphics and immersive 3D environment of VR can possibly address the limitations of traditional teaching methods when it comes to complex pharmacology topics.

Literature suggests that VR improves both knowledge and skills in medical students in comparison to traditional educational methods [4,5]. Moreover, VR offers additional benefits such as increased engagement, confidence, and retention, while reducing cognitive loads among learners [8]. Despite the potential advantages, there is limited research on the application of VR technology in enhancing pharmacology learning outcomes.

Given the considerable investment of resources and time required for implementing VR in the curriculum, it is crucial to determine its suitability for pharmacology education, its effectiveness in increasing learners' knowledge, and its acceptance among learners. Therefore, the aim of this pilot study was to investigate the impact of VR on pharmacology learning and to assess learners' perception of VR as an educational tool.

## Materials and methods

Development of VR learning module

A VR learning module on the pharmacology of selected cardiovascular drugs was created following Kern’s six-step framework: problem identification and general needs assessment, needs assessment of targeted learners, goals and objectives, educational strategies, implementation, evaluation and feedback [9]. Based on the needs assessment of students at the University of Illinois College of Medicine at Peoria (UICOMP), the VR learning module focused on the pharmacology of cardiovascular drugs associated with the autonomic nervous system. The needs assessment included student survey and consensus of pharmacology faculty.

The VR learning module was based on the following learning objectives:

1. List the various types of autonomic receptors present on heart and blood vessels, and describe the effects of agonists and antagonists.

2. Explain the effect of each class of autonomic drugs on systolic and diastolic blood pressure, peripheral vascular resistance, and heart rate.

To effectively demonstrate the effects of drugs, such as adrenergic agonists and antagonists at both the cellular (e.g., receptor subtypes) and organ levels (e.g., cardiac contractility and vascular resistance), 2D and 3D animations were created. The digital assets were developed at the Jump Simulation Center (Peoria, USA). The learning module was developed using HTC Vive Pro HMD (resolution: 1440x1600 pixels, refresh rate: 90 Hz) and Enduvo software (Peoria, USA). The module incorporated an instructor-guided mode and a self-paced interactive learning mode in an immersive environment.

Participants

Medical students from first to fourth years, enrolled at the UICOMP, were invited to participate in the study. The autonomic nervous system and relevant cardiovascular drugs are included in the first block of M1 curriculum. All participants had the same level of baseline knowledge of the cardiovascular drugs and receptor interactions covered in the VR module. The study was approved by the institutional review board (approval #1889070-1) at UICOMP in 2022. Informed consent was obtained from all participants.

Study design

A prospective mixed-methods design was used for this study. The goal of the current study was to investigate the impact of VR without direct comparison to traditional learning. Therefore, we adopted a single group pre-test-post-test design, omitting the need for a traditional learning control group. Both tests were comprised of five multiple-choice questions related to the pharmacology concepts covered in the module. Additionally, participants provided self-ratings of their pharmacology knowledge on a five-point Likert scale (1 = poor; 5 = excellent).

**Figure 1:**
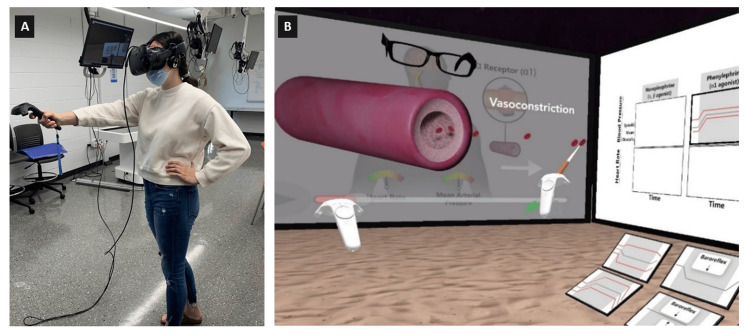
(A) A study participant is interacting with the VR environment using a HMD and controller; (B) The VR interface of the cardiovascular pharmacology module VR: Virtual reality; HMD: Head-mounted display

We used Kolb’s experiential learning model as a framework to design the learning experiences in VR. According to this model, knowledge acquisition is increased when learners cycle through four phases, which are: concrete experience, reflective observation, abstract conceptualization, and active experimentation [10]. Before engaging with the VR module, each participant received a brief tutorial on how to use the VR platform and HMD with hand-held controllers (Figure 1A). Subsequently, they were given access to the VR module for 10 minutes in the laboratory (Figure 1B). The participants observed different scenarios on drug actions in the VR module and at the end of the module they were given a task to match relevant patient charts with particular drugs.

To evaluate attitudes and perceptions, a Qualtrics® survey was designed with a five-point Likert scale to assess learner satisfaction, ease of use, perceived usefulness, quality of visual elements, intention to use, and comfort level (1 = strongly disagree; 5 = strongly agree). In addition, participants were invited to provide feedback through an open-ended question.

Data Analysis

The data was downloaded from the Qualtrics® platform and analyzed using GraphPad Prism (San Diego, USA). A two-tailed t-test was performed to assess the significance of any observed differences between pre- and post-test scores. The difference between scores was considered significant at p <0.05. Results were expressed as mean ± SD.

## Results

A total of 32 medical students (M1, n = 9; M2, n = 15; M3; M4, n = 8) participated in the study. The age range of the majority of participants was between 20 to 29 years (male, n = 14; female, n= 18).

The mean scores of pre- and post-intervention tests were statistically different (p <0.005) (Table 1), indicating VR module significantly improved knowledge acquisition in study participants. Likewise, the mean scores of the self-rated knowledge level between pre- and post-intervention were statistically different (p <0.005) (Table 1), indicating VR module increased confidence in pharmacology. The knowledge test scores exhibited no significant difference between students of varying levels (data not shown).

**Table 1:** The pre- and post-VR exposure scores of knowledge test and self-rated knowledge level VR: Virtual reality

	Knowledge Test	Self-Rated Knowledge Level
Pre-scores (mean ± SD)	3.31 ± 1.1	2.47 ± 1.0
Post-scores (mean ± SD)	4.16 ± 1.1	3.25 ± 1.0
P-value	<0.005	<0.005

The post-intervention survey demonstrated high internal reliability, with a Cronbach’s alpha value greater than 0.8. The quantitative survey data is presented in Table 2. According to the survey results, 90% of the participants expressed satisfaction with the VR module. Moreover, 90% of the participants found the VR technology easy to use and believed that their time was efficiently utilized. However, a minority of participants (15%) indicated a preference for the traditional learning format, and some participants (20%) reported experiencing discomfort while using VR.

**Table 2:** Participants’ perception of their experience with VR. Five-point Likert scale was used where 1 = strongly disagree and 5 = strongly agree. VR: Virtual reality

Item	Mean ± SD
The quality of the pharmacology learning module was adequate.	4.45 ± 0.8
The material presented in the pharmacology learning module adequately addressed the learning objectives.	4.50 ± 0.8
The technology used for the pharmacology learning module was easy to use.	4.60 ± 0.8
The pharmacology learning module made it easier to review the concepts.	4.50 ± 0.9
I would have preferred a presentation in a traditional learning format.	2.65 ± 0.9
The pharmacology learning module made me more confident to apply this knowledge in a clinical setting.	4.15 ± 0.8
My time was efficiently used on the pharmacology learning module.	4.50 ± 0.9
I would recommend my peers to use this pharmacology learning module.	4.25 ± 1.1
The pharmacology learning module caused feelings of discomfort that adversely impacted my ability to learn content.	1.80 ± 1.2
Overall, I was satisfied with this pharmacology learning module.	4.45 ± 0.8

In response to the open-ended question for additional comments/suggestions, the majority of the participants showed a positive attitude towards the VR experience. Some of the representative comments were as follows:

“I think this was useful and much more innovative than our traditional learning modules.”

“I loved this experience! Very engaging for people who tend to wander off during lectures.”

However, some comments expressed mixed reactions toward VR. Some of the representative comments were as follows:

“I did not like the feeling of the VR helmet on my face/head while trying to learn. I think VR offers a way to learn without distractions such as my phone, but I would prefer a traditional video format.”

“Being able to go at my own pace made this more valuable to me than a traditional lecture :) I did get a little bit dizzy but I can go at my own pace so I can take the headset off.”

## Discussion

The use of VR in pharmacology education is a relatively novel concept compared to its application in other disciplines. While some pharmacology educators in medical or pharmacy programs have experimented with VR content for learners, there is a lack of evidence regarding its effectiveness [11-13]. This pilot study is an attempt to evaluate the effectiveness of VR in pharmacology education using a mixed methods design. Our findings suggest that VR-based learning significantly improved pharmacology knowledge and confidence levels in medical students.

Our findings align with a previous study that demonstrated increased pharmacology knowledge acquisition in nursing and midwifery students using a Cave Audio Visual Experience (CAVE) system [14]. The CAVE system creates a large-scale semi-immersive 3D environment through multiple liquid-crystal display (LCD) panel projections arranged in a 320-degree enclosure [14]. In contrast, we used HMDs in our study. HMDs produce a higher level of spatial orientation than CAVE [15]. With the significant reduction in the cost of HMDs in recent years, they are now more accessible for learners and more likely to be adopted by educational institutions. HMDs are comparatively less expensive than CAVE. The cost of VR equipments (HMD, controllers, and PC with graphics processing unit) that were used in each VR station of our study was around $3000, whereas an entry-level CAVE system costs more than $50,000 [3].

The questionnaire results revealed that the majority of the participants responded positively to this innovative learning tool. Comments from participants reflected their enjoyment of the VR experience, noting that it provided a distraction-free environment with self-paced learning opportunities. While most of the participants were satisfied with VR learning, many did not view it as a complete replacement for traditional pedagogy. Instead, VR was viewed as a valuable supplementary educational tool to enhance pharmacology learning, complementing traditional teaching methods. 

One of the drawbacks associated with VR is the occurrence of cybersickness, with symptoms like headaches, dizziness, blurred vision, and motion sickness [16]. To address this concern, we restricted the VR experience to 10 minutes for each participant. While the majority of the participants reported no discomfort with VR, one student mentioned experiencing dizziness in the comment.

Limitations 

Our research has several limitations. First, the data was collected from a single institution and involved a relatively small sample size, which may limit the generalizability of our findings to other institutions. Second, the participants in our study were volunteers rather than randomly selected from the student body, which introduces the possibility of bias in the study results.

## Conclusions

In conclusion, our findings suggest that VR is effective in increasing knowledge and confidence levels among medical students. The positive feedback from the students, highlighting the distraction-free and self-paced environment provided by VR, further reinforces its potential as an innovative educational tool for pharmacology. Specifically, VR appears to be highly beneficial in covering complex pharmacological concepts and drug actions, making it a valuable tool to complement traditional methods used in pharmacology education. Further research and implementation of VR in medical curricula are needed to enhance student's learning experiences and ultimately improve their understanding of pharmacology.
